# Colon cancer patient with long-term colon stent placement: Case report and literature review

**DOI:** 10.3389/fonc.2022.972454

**Published:** 2022-08-23

**Authors:** Qing Huang, Min-hong Zou, Wen-long Liang, Jian-chang Wei, Jie-feng Xie, Yong-Qiang Li, Wang-lin Li, Jie Cao

**Affiliations:** ^1^ Department of Gastrointestinal Surgery, Guangzhou Digestive Disease Center, Guangzhou First People’s Hospital, the Second Affiliated Hospital of South China University of Technology, Guangzhou, China; ^2^ Department of Ultrasound, the Third Affiliated Hospital, Sun Yat-Sen University, Guangzhou, China; ^3^ Department of Gastroenterology and Hepatology, Guangzhou Digestive Disease Center, Guangzhou First People’s Hospital, the Second Affiliated Hospital of South China University of Technology, Guangzhou, China

**Keywords:** colon cancer, colon stent, radical resection, elderly, self-expandable metal stent (SEMS)

## Abstract

Colorectal cancer (CRC) is the third most common cancer and the second leading cause of cancer mortality globally. Large bowel obstruction (occurring in 15-30% of patients with CRCs) accounts for approximately 80% of medical emergencies related to CRC. Currently, there is no standard treatment of this condition. The European Society of Gastrointestinal Endoscopy (ESGE) recommends self-expandable metal stent (SEMS) as a bridge (two weeks) to surgery for left-sided obstructing colon cancer. In the present report, we describe an 81-year-old male with colon cancer who underwent colon stent placement for 32 months, but later underwent radical resection. A follow-up of more than four-months revealed that his condition was normal. The history as well as application and advantages of SEMS are discussed in this report.

## Introduction

Colorectal cancer (CRC) is the third most commonly diagnosed cancer, with about 1.9 million new cases reported each year. It is the second leading cause of cancer death in the world, with almost 935,000 deaths recorded in 2020 (1). Large bowel obstruction (LBO, occurring in 15–30% of CRC) accounts for almost 80% of all emergencies related to CRC (2). About 75% of cases of obstruction in CRCs occur in the distal and splenic flexure segments, and the most common location is the sigmoid colon (3). Obstructive right-sided colon cancer is usually treated through an emergency surgery with primary resection and ileocolic anastomosis (4). However, it is not clear whether emergency surgery should be performed for obstructive left colon cancer (OLCC), because emergency surgery is associated with the substantial colostomy rates and mortality rates, especially in elderly patients. Currently, elective placement of a self-expandable metallic stent (SEMS) is performed to prevent the obstruction. This is applied either as a palliative treatment (PAL) in incurable disease (not amenable to colectomy or colostomy) or as a bridge to surgery (BTS) in patients with potentially resectable CRC (5, 6).

Placement of SEMS was first described in 1991 by Dohmoto (7). Since then, the use of SEMS for BTS has yielded inconsistent results in terms of stent complications, locoregional recurrence, disease-free and overall survival rates, among other parameters. Application of SEMS as BTS has fewer surgical complications, low duration of hospital stays and intensive care unit, reduces costs, as well as better primary anastomosis compared with emergency surgery. Therefore, SEMS are safe and highly effective in the short-term management of colorectal obstructions, especially in frail elderly patients (8). In another study, it was found that colonic SEMS as a bridge to surgery resulted in better long-term oncologic outcomes compared with direct surgery (9). The European Society of Gastrointestinal Endoscopy (ESGE) recommends SEMS as a bridge to surgery for OLCC and suggests a time interval of approximately 2 weeks before resection (5). Here, we report a case of colon cancer patient who underwent SEMS placement for 32 months, and did not develop distant metastasis. The patient later underwent radical surgery.

## Case report

**Figure 1:**
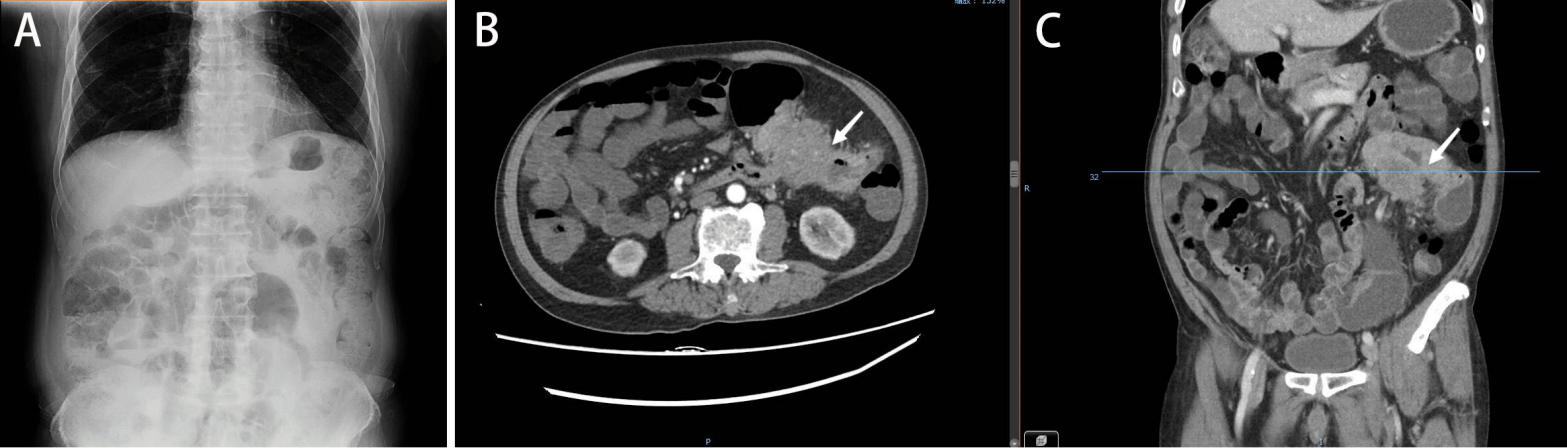
**(A)**. Plain abdominal radiograph showing intestinal obstruction. **(B, C)** Abdominal CT illustrating colon cancer causing obstruction (June 2019).

**Figure 2:**
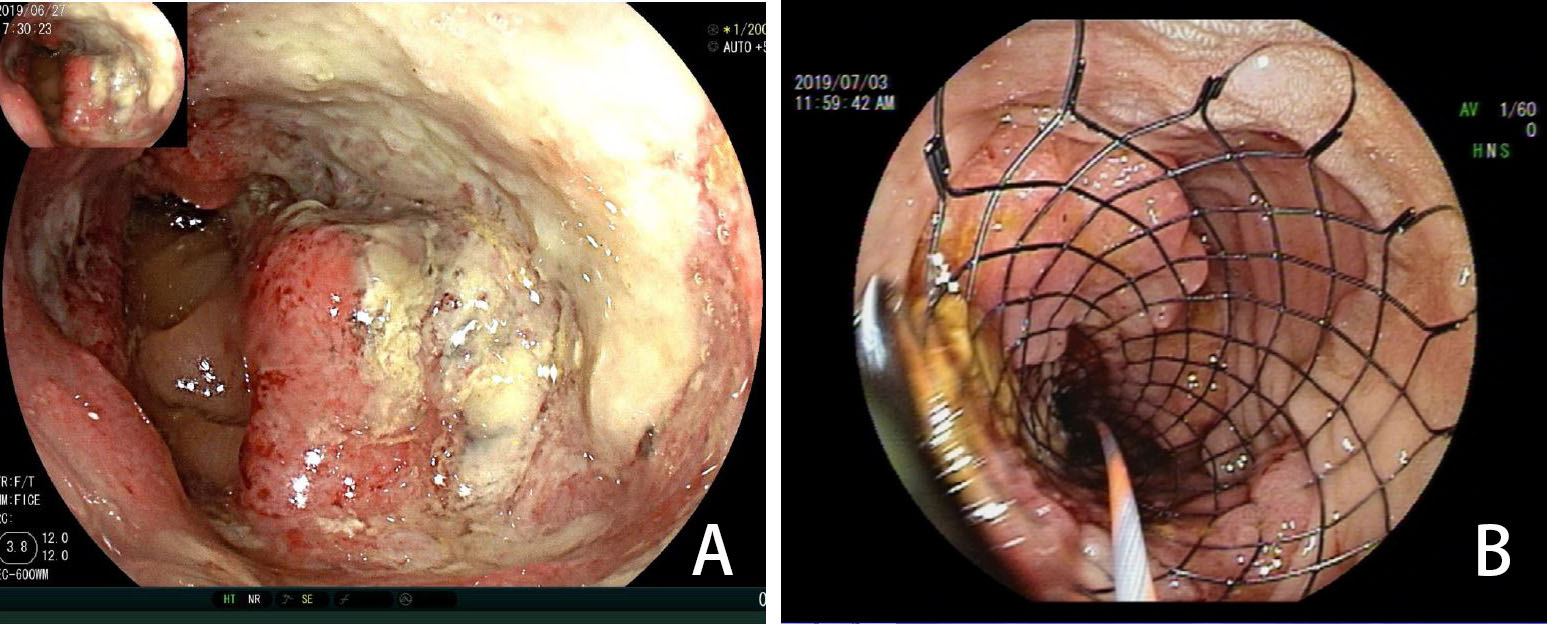
**(A)** Colonoscopy showing a tumor (June 2019). **(B)** Colonoscopy showing a stent placed to prevent obstruction (July 2019).

**Figure 3:**
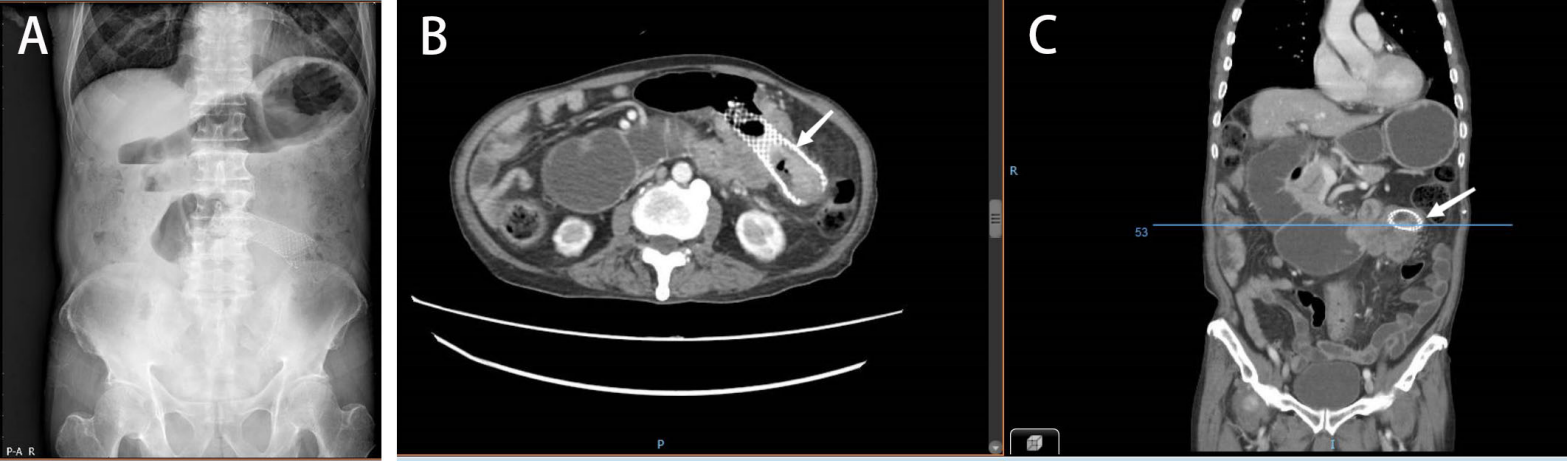
**(A)** Abdominal plain radiograph showing the duodenal obstruction. **(B, C)** Abdominal CT demonstrating a tumor invading the duodenum and causing duodenal obstruction due to compression (January 2022).

**Figure 4:**
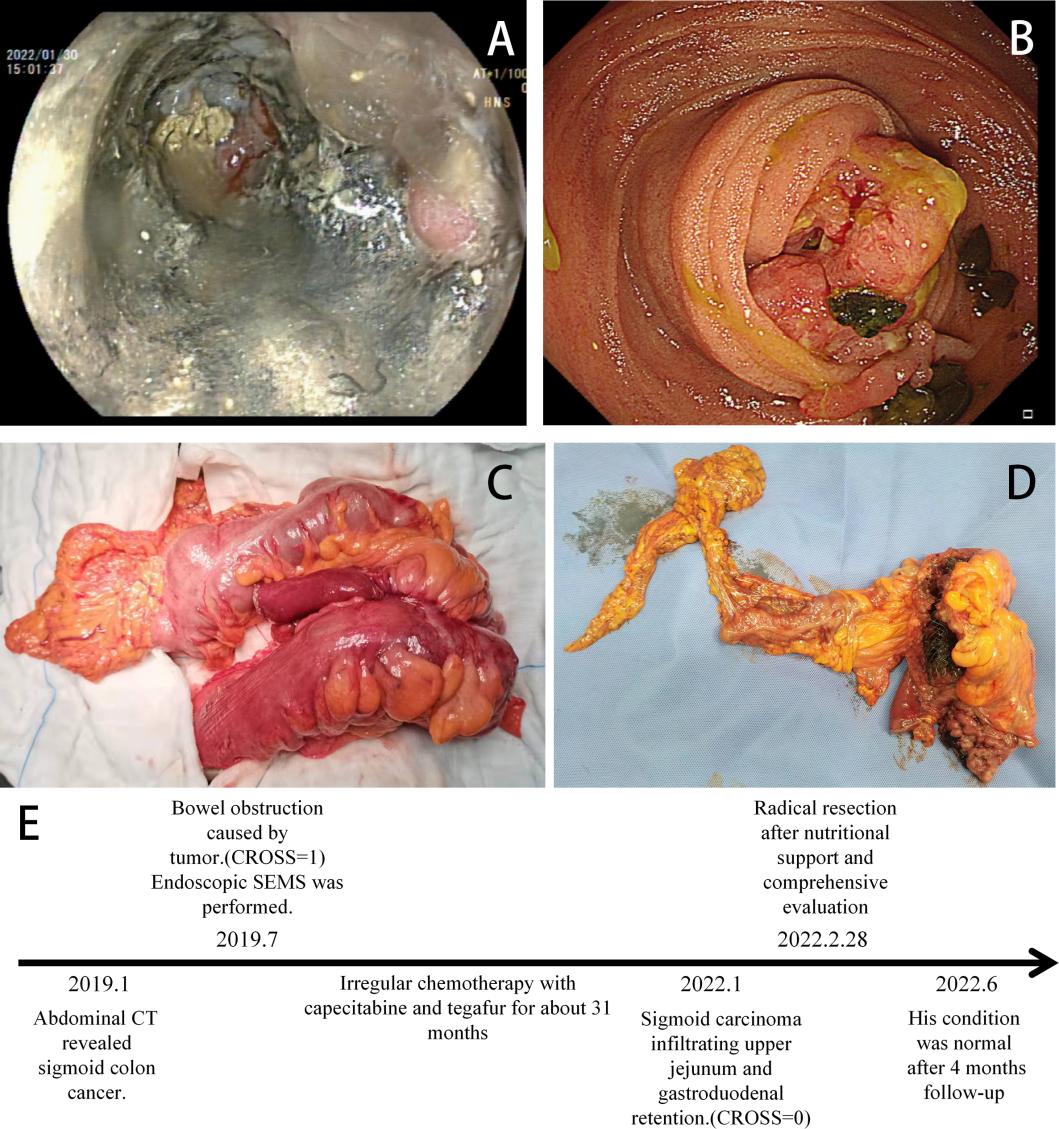
**(A)** Colonoscopy indicating stent tumor necrosis. **(B)** Gastroscopy showing a tumor invading the duodenum (January 2022). **(C)** Intraoperative tumor (The jejunum and duodenum are severely affected). **(D)** Postoperative tumor incision specimen (February 28, 2022). **(E)** Timeline of the relevant treatments.

An 81-year-old male patient was hospitalized due to cerebral infarction in January 2019. The patient had a history of hypertension and gout. Abdominal CT examination revealed sigmoid colon cancer. The patient was advised to undergo colonoscopy to confirm the diagnosis, but his family refused this test. Six months later, the patient was hospitalized due to repeated abdominal pain, bloating, and inability to eat. A diagnosis of bowel obstruction caused by sigmoid colon tumor was made (**Figure 1**). Colonoscopy revealed moderately differentiated adenocarcinoma (**Figure 2A**). The ColoRectal Obstruction Scoring System (10)(CROSS) was 1 and the concentration of carcinoembryonic antigen (CEA) was 311.9 ng/ml. The patient’s chest and abdomen CT showed that the tumor stage was T4N2M0. On July 2019, endoscopic SEMS was performed (**Figure 2B**). It was recommended that the patient undergo surgical treatment 2 weeks later. However, his family was hesitant to the surgery. Therefore, he was put on irregular chemotherapy with capecitabine and tegafur for about 31 months. In January 2022, the patient was hospitalized for repeated vomiting, inability to eat and walk. His CROSS score was 0, and assessment using the nutritional risk screening 2002 showed that the patient was a high-risk category. The concentration of CEA was 530ng/ml. Abdominal plain radiograph and abdominal CT revealed a sigmoid carcinoma infiltrating upper jejunum and gastroduodenal retention (**Figure 3**). Colonoscopy showed that the SEMS was blocked preventing entry of endoscope (**Figure 4A**). Gastroscopy revealed a mass blocking the bowel in the horizontal segment of the duodenum, and pathological biopsy confirmed a diagnosis of adenocarcinoma (**Figure 4B**). The patient received several treatments but his condition did not improve. A multidisciplinary team recommended surgery for the patient to which his family agreed. The patient received parenteral nutritional support for one month and received a preoperative comprehensive evaluation of operative tolerance, postoperative complications, physical and mental ability, and family support. Although most physicians recommend palliative surgery (bypass surgery) based on the patient’s age and underlying disease, we suggested radical resection on 2022-2-28 as follows sigmoid colectomy + lymph node dissection + partial resection of duodenum and upper jejunum + duodenojejunostomy + descending colostomy (**Figures 4C, D**). After surgery, the patient was transferred to the intensive care unit (ICU) for further treatment. During ICU, the patient developed hyperkalemia and oliguria, which were treated with continuous renal replacement therapies (CRRT), along with anti-infection and nutritional support. The patient also received early enteral nutrition (on the second day postoperatively) because the intraoperative enteral feeding tube was placed 30 cm distal to duodenojejunal anastomosis. One week later, he was transferred to the general ward to continue enteral nutrition, rehabilitation, and other treatments. The patient was discharged after 2 weeks. At discharge, the patient could eat semi-fluid foods and walk with assistance. Pathological findings were as follows: T4bN2aM0, IIIC stage, lymph node positive 5/21, MSS, BRAF-, nerve bundle invasion, and no vascular invasion. There was also no evidence of local recurrence and distant metastasis during 4 months follow-up. A timeline diagram of the relevant treatments is shown in **Figure 4E**. The CARE checklists were provided in Supplementary File (11).

## Discussion

Colorectal cancer (CRC) is one of the most common cancers worldwide and large-bowel obstruction caused by advanced colonic cancer occurs in 15-30% of patients with colon cancer (2, 12). Previous studies have shown that more than half of acute obstructive colon cancer occurs on the left side, most commonly in the sigmoid colon (3, 13). The anatomic characteristic of a narrow luminal diameter in the left-sided colon explains the higher incidence of obstructive colon cancer compared with the right-sided colon.

Currently, there is no standard management protocol for this large-bowel obstruction due to CRC, especially for OLCC. Traditionally, primary oncologic resection *via* right hemicolectomy or extended right hemicolectomy with ileocolic anastomosis has been advocated for right-sided obstructive colon cancer (4). The appropriate surgical management for OLCC is still a matter of debate and several options have been proposed. There are generally two strategies to treat a primary tumor when the tumor is resectable. The first one is the primary resection [primary anastomosis (RPA) and Hartmann’s procedure (HP)], and the other is the second stage resection (the first stage includes simple stoma, transanal decompression tube and colon stent). Segmental resection as a mode of RPA, which can reduce the patient’s stoma, but it results in higher anastomotic leakage compared with HP,

this shortcoming limits its application. To prevent higher anastomotic leakage, subtotal colectomy (SC) or total colectomy (TC) have been proposed. Major disadvantages of SC and TC include prolonged operative time and poor colonic function, with many patients having an increased risk of developing electrolyte disturbance because of diarrhea (14). If the obstruction causes right colonic ischemia, caecal tears or perforation, or if synchronous proximal malignant tumors are present, these operations (SC and TC) are recommended (15). Although HP does not increase the risk of anastomotic leakage, it results in a high risk of mortality following emergency surgery, and long-term ostomy (16). In elderly patients, the mortality rate following emergency surgery is high due to the potential underlying diseases and low surgical tolerance. For two-stage procedure, simple ostomy as the first stage can relieve the patient’s obstructive state and tumor resection is performed in the second stage, the cumulative mortality and complication rates of these two operations are not lower than those of HP (17). Meanwhile, patients with the two-stage procedure need to tolerate the trouble of stoma, and the long waiting time without resection of the primary tumor may lead to tumor progression. A retrospective study revealed permanent stoma rate and intermediate-term oncologic outcomes did not significantly differ when the simple ostomy or SEMS as a bridge to surgery for OLCC (18). It has been proposed that tube decompression of acute colonic obstruction is an easy and cost-effective operation as it reduces the risk of emergency operation with all their sequelae (19, 20). Although this method reduces the risk of surgery, transanal decompression tube (TADT) is prone to blockage and does not effectively relieve the obstruction when compared with colon stents. Kawachi et al. found SEMS as BTS for OLCC seems to be effective in avoiding permanent stomas than TADT (18). Moreover, the 3-year relapse-free survival rate of TADT group is lower than that of SEMS (21).

Since its first description in 1990s, SEMS has been widely used in clinical practice. The 2017 WSES and ESGE guidelines concluded that SEMS decompression as BTS offers a better short-term outcome compared with immediate emergency surgery (4, 5). A previous study showed that application of SEMS as BTS for OLCC seems to be an oncologically safe alternative to emergency resection (22). SEMS as a BTS for OLCC was associated with lower short-term overall morbidity and lower rates of temporary and permanent stoma (23), and which can reduce in-hospital death and medical costs compared with emergency surgery (24). Recent studies have shown that adoption of SEMS as BTS yields favorable long-term oncological outcomes when compared with direct emergency surgery (9, 25). The cohort study reported satisfactory long-term outcomes of SEMS as BTS, which was ascribed to a reduced perforation rate (26). The meta-analysis showed SEMS as BTS was oncologically safe when in experienced hands (27). Therefore, ESGE recommend SEMS as a bridge to surgery, and as an alternative treatment to emergency resection for patients with potentially curable OLCC (5).

Clinical studies have reported different technical success rates (90.5%-98%) of SEMS stent placement (6, 28–31). In our center, the success rate is close to 96%. Technical success was defined as accurate SEMS placement, conferring adequate stricture coverage on the first attempt, free of procedure-related adverse events, such as perforation, re-obstruction, stent migration, infection/fever, abdominal pain, and tenesmus (6). The technical difficulty of SEMS was significant association with the CROSS score and stricture length (6). In this case, the CROSS before stent placement was 1, the length of stent placement was 9 cm, the implantation process was smooth (**Figure 2B**). The placement of the SEMS stent enabled the patient to eat semi-liquid food. ESGE suggests a time interval of approximately 2 weeks before resection when SEMS is performed as BTS in patients with curable OLCC. A study by Sato et al. showed surgery within 16 days after stenting may increase the benefit of SEMS without interfering with short- and long-term outcomes (32). Other studies have reported a cutoff value of 15 days to be ideal for reducing the risk of postoperative complications (33, 34). SEMS as BTS followed by neoadjuvant chemotherapy before elective surgery is a safe and well-tolerated approach in patients with OLCC (35). In this case, the patient was advised to undergo surgery after 2 weeks. However, the patient underwent surgery after 32 months of implantation due to reasons given by the patient and his family. The stent patency of the patient was maintained for 32 months, but the tumor had only local infiltration (infiltration into the duodenum, **Figure 4B**) and no distant metastasis. On the one hand, it is benefited from the slower invasion process of colon cancer in the elderly, on the other hand, the smoothness of the stent ensures the nutritional status of the patient, and the patient’s irregular chemotherapy slows down the tumor invasion speed of the patient.

After stent placement, laparoscopic treatment or open surgery can performed. Studies have shown that laparoscopic surgery is safe and effective (36–38). In this case, due to the duodenum local infiltration, the patient developed obstruction with a score of 0. Considering the patient’s age and existence of underlying diseases, open surgery was performed instead of laparoscopic surgery. The tumor and the infiltrating duodenum were completely resected during surgery. Early enteral nutrition (jejunal feeding tube placed during operation) and early postoperative CRRT were implemented in the ICU to provide postoperative nutritional support, reduce infection, cardiopulmonary complications, and improve renal function. The patients showed good recover after this treatment.

More than 60% of CRC patients are >70 years old (39), the majority of such patients show an obstruction at the initial discovery (12). SEMS provide a bridge for surgical operation and provide an alternative option for palliative care for these patients. In China, elderly patients tend to follow the advice of family members regarding their treatment. However, in some cases, the treatment advises given by family members and physicians are biased. In this case, the patient’s family refused the doctor’s recommended treatment two times. Nevertheless, palliative surgery was recommended based on the patient’s age. One of the major challenges with radical surgery is the physiological heterogeneity of the older patient population. There are discrepancies between physiological and chronological ages. Moreover, coexisting medical conditions, as well as psychological and social care issues are diverse among elder patients (39). If it is only considered chronological age and life expectancy, the treatment plan for elder patients maybe undertreated. The International Society of Geriatric Oncology (SIOG) recommended that elderly CRC patients requiring surgery should undergo a preoperative whole patient evaluation for the most common physiological side-effects of aging, physical and mental ability, and social support (8, 39). Therefore, comprehensive assessments and individualized treatment approaches should be performed for elderly patients with CRC (8, 39).

In the conclusion, SEMS may serve as a bridge to surgery of OLCC, with better short-term and long-term outcomes compared with emergency resection. ESGE recommends a time interval of approximately 2 weeks before resection, the longer interval time may benefit these patients with OLCC, and chemotherapy during this time interval can have played an important role. However, more clinical studies are needed to prove this deduction. For elderly CRC patients, comprehensive assessments and individualized treatment approaches should be performed before surgery.

## Data availability statement

The original contributions presented in the study are included in the article/supplementary material. Further inquiries can be directed to the corresponding authors.

## Ethics statement

Written informed consent was obtained from the individual(s) for the publication of any potentially identifiable images or data included in this article.

## Author contributions

Guarantors of integrity of entire study: HQ, MZ, WLiang, WLi, JC; study concepts/study design or data acquisition or data analysis/interpretation: all authors; manuscript drafting or manuscript revision for important intellectual content: all authors; approval of nal version of submitted manuscript: all authors; literature research: MZ, QH; and manuscript editing: all authors. All authors contributed to the article and approved the submitted version.

## Funding

Guangzhou Planned Project of Science and Technology (202102010024); Guangdong Natural Science Foundation (2021B1515420004) Special Clinical Technology Program of Guangzhou, China (2019TS52); Science and Technology Projects in Guangzhou (202201020363). The program of Guangdong Provincial Clinical Research Center for Digestive Diseases (2020B1111170004); Guangzhou High-level Key Clinical Specialty Construction Project (No. 9); The Project of Key Medical Discipline in Guangzhou (2021-2023).

## Conflict of interest

The authors declare that the research was conducted in the absence of any commercial or financial relationships that could be construed as a potential conflict of interest.

The reviewer ZY declared a shared parent affiliation with the author MH-Z to the handling editor at the time of review.

## Publisher’s note

All claims expressed in this article are solely those of the authors and do not necessarily represent those of their affiliated organizations, or those of the publisher, the editors and the reviewers. Any product that may be evaluated in this article, or claim that may be made by its manufacturer, is not guaranteed or endorsed by the publisher.
